# Impact of COVID-19 on health-related behaviours, well-being and weight management

**DOI:** 10.1186/s12889-021-11143-7

**Published:** 2021-06-16

**Authors:** Amanda Avery, Josef Toon, Jennifer Kent, Laura Holloway, Jacquie Lavin, Sarah-Elizabeth Bennett

**Affiliations:** 1 The University of Nottingham, School of Biosciences, Sutton Bonington campus, Loughborough, LE12 5RD UK; 2 Slimming World, Alfreton, Derbyshire England DE55 4SW

## Abstract

**Background:**

Weight management is complex for people even in times of stability. Supporting individuals to develop strategies to maintain a healthier weight when there are additional life challenges may prevent relapse. This mixed-methods study describes the impact the COVID-19 restrictions had on adults engaged in weight management before and during the pandemic in order to determine helpful strategies.

**Methods:**

Longitudinal data was captured from online surveys completed by Slimming World (SW) members 0–4 weeks after joining, October/November 2019, providing pre-joining and baseline (T0&T1), 3- (T2) and 6- month (T3-during COVID-19) data. Representatives from the general population, not attending a weight management service, completed the same questionnaires providing cross-sectional control data. All weights are self-reported. For this study, questions assessing the impact of the COVID-19 challenges on health-related behaviours and well-being are included comparing responses at T0/T1, T2 & T3. Longitudinal data were analysed using repeated measures ANOVA and cross-sectional data, one-way independent ANOVAs to compare means. Comparisons between SW members and controls were determined using z-proportion tests.

Qualitative data generated was thematically analysed using a six-step approach to produce the key emerging themes.

**Results:**

222 SW members completed all three surveys, achieving a weight loss of 7.7 ± 7.5%. They maintained positive health-related behaviour changes made since joining, including increased fruit and vegetables (*p* < 0.001), fewer sugary drinks (*p* < 0.001), cooking from scratch (*p* < 0.001) and increased activity levels (*p* < 0.001). Despite COVID-19 restrictions, they were still reporting improvements in all behaviours and had healthier scores than the controls on all but alcohol intake, although still within guidelines. Qualitative data indicated that the situation created various challenges to managing weight with fresh foods harder to access, comfort eating, drinking more alcohol, eating more sugary foods and snacking through boredom. However, some reported having more free time enabling better planning, more time to cook from scratch and increased physical activity.

**Conclusions:**

The findings highlight the value of peer, group and online support and guidance for individuals to develop sustainable behaviour changes and a level of resilience. These strategies can then be drawn upon enabling maintenance of lifestyle changes and management of weight even in challenging times.

## Introduction

The COVID-19 pandemic is a global health and human crisis threatening the food security and nutrition of many people. It has become increasingly acknowledged that obesity increases the severity of symptoms and impairs treatment outcomes in those affected by COVID-19 [1]. Before the COVID-19 pandemic, different levels of malnutrition were already prevalent with many people consuming an energy dense, nutrient poor diet [2]. The majority of the UK population were not participating in recommended levels of daily activity for physical and mental health benefit and to support maintenance of a healthy weight [3]. Weight control is complex, influenced by an interaction of various factors including biological, behavioural, environmental, societal and cultural [1]. People are faced with many challenges towards successful, sustainable weight control and relapse is common even when their lives are relatively stable and there is some degree of certainty. People benefit from support to develop personal and practical strategies which can become part of their routine and help them to lose weight and maintain their weight in the long term [4, 5]. Many people value support to feel empowered to develop these strategies to draw upon at times of challenge. The COVID-19 pandemic added to difficulties in the ability to maintain many health-related behaviours with the need for some people to shield, limitations to opportunities to be physically active alongside reports of changes in shopping and cooking habits, access to foods, changes to dietary intake such as decreased consumption of fresh fruit and vegetables, comfort eating, increased alcohol purchasing and increased snacking on cakes, biscuits, confectionary and savoury snacks for example [1, 6]. Many of which may add to the complexities of maintaining a healthier weight during the UK first lockdown period.

Slimming World is a UK based weight management organisation, which uses a multi-component approach focussing on psychological as well as physiological aspects of weight control to encourage the formation of new healthy eating habits and increases in physical activity levels. The underpinning psychological approach is built on a number of behaviour change techniques including self-determination theory and the commitment model, but essentially offers positive support and reinforcement, with no criticism or judgement. It also focuses on supporting members to become less self-critical and more self-compassionate, helping them to cope with set-backs throughout their weight loss journey and arming them with the skills needed for successful long term weight control. Slimming World’s Health and Well-being study is an ongoing piece of work surveying new adult members, who joined the weight management programme between October and November 2019, at regular time points over a 12-month period. Data is obtained about various aspects of health-related behaviours and well-being including dietary habits, eating behaviours, physical activity levels, alcohol intake and mood. A comparator survey of a representative general population sample is captured at each time point. The six-month time point coincided with first ‘lockdown’ resulting from the COVID-19 pandemic. Hence the study team added additional questions with the aim to capture how the lockdown restrictions might have impacted on the various aspects of health-related behaviours and well-being being monitored through the study. The hypothesis was that the lockdown restrictions negatively impacted on health behaviours related to weight management and also to people’s sense of well-being, but with weight management support reducing the level of negative impact. This paper reports the six-month findings with emphasis on the impact of the COVID-19 pandemic on weight management at this six-month follow up timepoint using the quantitative and qualitative data.

## Methods

New adult members, defined as those adults (≥18 years) joining Slimming World (SW) between 03/10/2019 and 07/11/2019 were invited to take part in an online survey within the first 4 weeks of their membership. An invitation was directly emailed to members accessing either the group support or online programme. Participants were told that the survey would ask questions about their health, well-being, physical activity and diet. The invitation was for members to take part in the baseline survey which would capture retrospective data from before they became a member and within the first 4 weeks after joining. Follow up surveys were sent to capture data 3 and 6 months later, with a 12-month follow up also planned. Those taking part in the survey were offered the opportunity to be entered into a prize draw after the completion of each survey to win a high street shopping voucher. The survey questions were managed via the survey-platform Qualtrics (Qualtrics, Provo, UT, USA). Qualtrics was also used to recruit representative samples from the general population, based on the typical age, gender and BMI of the Slimming World membership, to provide a comparator group at each time point. The comparator groups were not currently managing their weight with a commercial organisation.

### Ethics

The study was performed in accordance with the Declaration of Helsinki and approved by the University of Nottingham, School of Biosciences Ethics Committee No: SB1819/36.

### Questionnaire

The wider survey consisted of 70 core questions covering demographic information and self-reported height and weights, detailed questions about dietary habits, alcohol intake and eating behaviours, physical activity and general health and well-being for each time point (Additional files 1 & 2). For the purposes of this paper, questions relevant to the impact of the COVID-19 situation on health-related behaviours and well-being were included, such as fruit and vegetable consumption ‘How many portions did you eat yesterday?’ (with numerical response capped at 13+ for analysis purposes); the frequency of fatty and sugary food intake and the frequency of cooking from scratch ‘In a typical month how often do you do the following?’ (asked on a 5-point scale ranging from ‘Once a month or more’ to ‘Once a day or more’); the frequency of wholegrain and sugary drink intake ‘In a typical week how often do you do the following’ (asked on a 5-point scale ranging from ‘More than once a day’ to ‘Never/occasionally’); alcohol intake ‘How many alcoholic drinks do you generally consume each week?’ (split by type and volume of drink to allow units of alcohol to be derived for analysis purposes); total hours of moderate-intensity physical activity were calculated using the Sport England Active Lives questionnaire, (https://www.sportengland.org/know-your-audience/data/active-lives?section=method_behind_active_lives).

A well-being score was created by adapting items from the SF-36 to assess how often participants felt calm and peaceful, (item 1), had a lot of energy (item 2), felt downhearted and low (item 3) and had been a happy person (item 4), with three additional questions assessing how often; participants had been in a sociable mood (item 5), felt stressed (item 6) and felt anxious (item 7). All items were scored from 6 (“All of the time”) to 1 (“None of the time”). A score from the scale was calculated by summing scores from all items after reversing questions on negative affect (items 3, 6 and 7) to create a total well-being score (min = 7, max = 42) where a higher score indicated a more positive well-being.

The scale assessing well-being was tested for reliability in multiple ways. Average item-total correlation was high (r = 0.77) and split-half reliability showed a high internal consistency estimate (r = 0.84). Cronbach’s alpha analysis also showed the well-being scale had high internal consistency (*α* = 0.88) with no items identified as improving the scale if removed with high inter-item correlation (r = 0.52).

Given that the 6-month time point coincided with lockdown, additional questions were added to determine the impact of the COVID-19 situation on health-related behaviours and the ability to manage weight. Participants were asked whether certain lifestyle behaviours had changed due to the situation, and if so, the direction of this behaviour change. The sections with an additional COVID-19 question included those related to diet and eating habits, alcohol intake, physical activity and mood and well-being. Participants were also asked to rate on a scale of 1–5, with 1 being ‘Very difficult’ and 5 being ‘Very easy’, how they had found managing their weight during the COVID-19 situation. An additional, final open-ended question, ‘Please could you explain why you have found managing your weight easy or difficult during the COVID-19 situation’ provided more in-depth, qualitative findings.

This survey was completed between April 9th – May 16th 2020 and the results from this survey are reported in this paper.

### Participants

The study team aimed to recruit around 2000 SW members at baseline to ensure a suitable sample size was available for later analysis. In total, 1884 SW members completed the first survey of which 222 SW members completed all subsequent surveys. A sensitivity analysis of this sample showed variance tests would be sensitive enough to detect small differences in measures (sensitive up to η_p_^2^ = .016) at 95% power with an alpha of 0.05.

Samples from the general population were collected in parallel with a similar aim to recruit 2000 participants at survey 1 and 2. This was reduced slightly at survey 3 to reduce the sample size differences between SW members with data from 637 members of the general population collected at the third survey. As the T3 sample were going to be compared to SW members, a sensitivity analysis to understand what differences were going to be reliably detected between the 2 samples was performed showing that at 95% power and an alpha of 0.05 small effect sizes would be detected reliably (sensitive to d = 0.28).

### Data time points captured in this research paper

For clarity, retrospective data will be referred to as T0 (data only collected for SW members to determine their health-related behaviours before they joined SW, collected at the same time as T1 data), data collected within the first 4 weeks of membership as T1, 3-month data as T2 and 6-month data as T3.

### Statistical analysis

All data were collated and analysed within R statistical programming software (version 3.6.3). For the Likert data, data was checked for normality. For all data reported, where there was no significant divergence from normality, parametric analyses were undertaken. Longitudinal data from SW were analysed using repeated measures ANOVA *(or Friedman tests where data followed a non-normal distribution)* followed by post-hoc comparisons to test differences between time points within groups with adjustments to *p*-values for multiple comparisons performed using Bonferonni correction. For the cross-sectional data collected from the general population samples, one-way independent ANOVAs *(or Kruskal-Wallis tests where data followed a non-normal distribution)* were used to compare means for the sample cohorts followed with post-hoc tests. Data within text are presented as mean ± standard deviation and error-bars within figures represent upper and lower 95% confidence intervals. Proportion data between SW members and the general population were compared using z-proportion tests.

Significance levels are indicated as * *p* < 0.05, **p < 0.01, ***p < 0.001.

### Qualitative data analysis

The responses, provided by both the SW members and the control group, to the question ‘please, could you explain why you have found managing your weight easy or difficult during the COVID-19 lockdown period’ were thematically analysed. The six-step framework [7] (1. Becoming familiar with the data, 2. Generating initial codes, 3. Searching for the themes, 4. Reviewing themes 5. Defining themes and 6. Writing-up) was followed in this study. Key and supporting sub themes were identified independently by four different members of the research team with agreement on the final listing.

## Results

The T1 characteristics of the 222 SW members who completed all surveys are compared to all 1884 members who completed the survey at T1 and apart from age there were no differences (Table 1). They are also compared to the general population sample at T3 (*n* = 637) where differences in age, weight and BMI were observed (Table 1).

**Table 1:** Characteristics of the 222 SW members who completed the survey at T1 and T3 compared to all the SW members who completed the survey at T1 and the general population sample who completed the survey at T3 *(all respondents v. study population)*

	Data from T1			Data from T3		
	T1 SW members (*n* = 1884)	T1 SW members (*n* = 222)	*P* value^	T3 SW member T3 (n = 222)	T3 General population sample (n = 637)	P value T3 SW vs T3 GP
Gender (%female/ %male)	93.9%/6.12%	93.7%/6.19%	ns	93.7%/6.19%	92.1%/7.86%	ns
Age (years)	47.7 ± 13.34	51.8 ± 13.6	***	52.6 ± 13.65	47.4 ± 11.91	*
Weight (kg)	92.7 ± 19.68	94.2 ± 20.17	ns	87.0 ± 18.35	80.7 ± 19.87	*
BMI (kg/m2)	33.8 ± 6.65	34.7 ± 7.09	ns	31.7 ± 6.53	29.8 ± 6.48	**
Median Annual household income	£30,000–£39,000	£30,000–£39,000	ns	£30,000–£39,000	£30,000–£39,000	ns

### Weight data

**Fig. 1:**
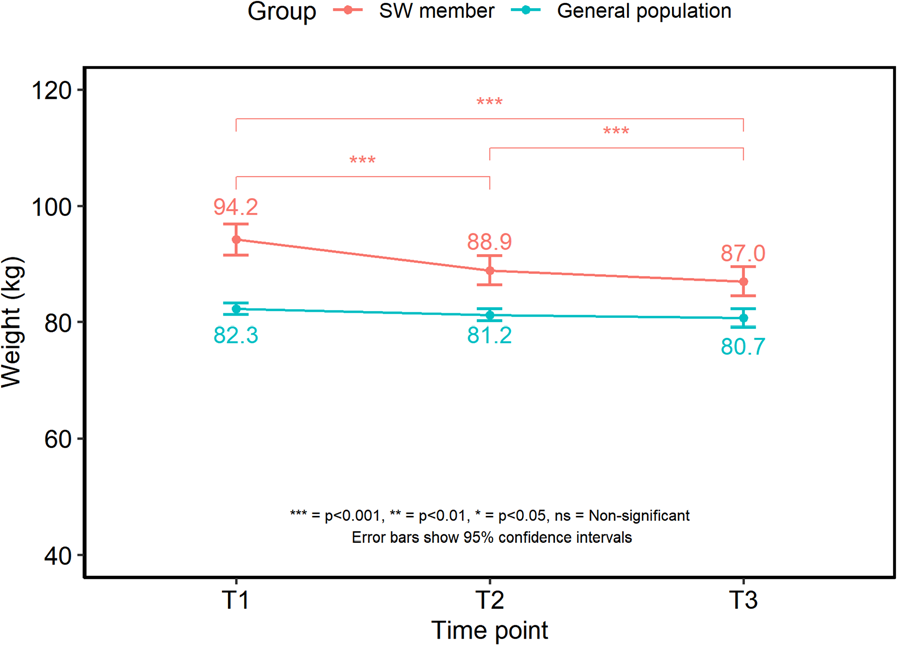
Mean and 95% confidence intervals for weight (kg) over time

Between the baseline and 6-month surveys (T1-T3), the 222 SW members achieved a mean weight loss of 7.7 ± 7.5% and a change in mean BMI of −3.0 ± 2.99 kg/m^2^. Weight changed significantly over time (F (1.25,276.78) = 161.209, *p* < 0.001), with post-hoc tests showing significant decreases by 3 months (94.2 ± 20.2 kg vs 88.9 ± 18.9 kg; T1 vs T2, *p* < 0.001) and between 3 and 6 month follow up points (88.9 ± 18.9 kg vs 87.0 ± 18.8 kg; T2 vs T3, *p* < 0.001). Weight did not differ significantly between each of the general population samples (Fig. 1).

### Health-related behaviour changes reflecting potential impact of COVID-19

#### Dietary changes (Fig. 2)

***Fruit intake*** Across the study time points, there was an overall significant change in fruit intake (F (2.87,577.23) = 64.15, *p* < 0.001), with post-hoc comparisons showing that SW members significantly increased fruit intake after joining (1.9 ± 1.34 vs 3.2 ± 1.40 portions; T0 vs T1; *p* < 0.001) and levels remained higher at T2 (3.1 ± 1.34 portions, *p* < 0.001) and T3 (3.0 ± 1.56 portions, *p* < 0.001).

**Fig. 2:**
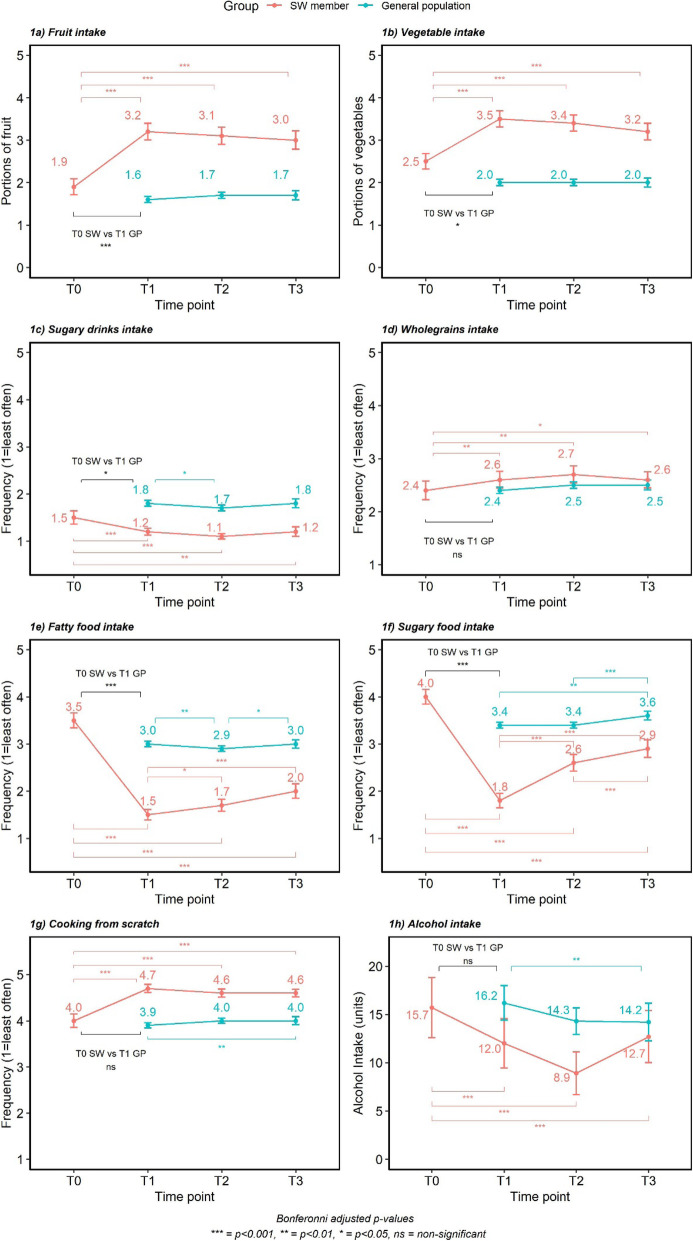
Mean changes and 95% confidence intervals for dietary behaviours reported over the 6-month period

For the general population, there was no significant difference in average daily fruit portions consumed between samples at each time point (F (2,3545) = 1.863, *p* > 0.05), with SW members eating significantly more portions of fruit at each time point (*p* < 0.05).

Data from the COVID-19 specific question showed that the majority of SW members and the general population reported their fruit intake had not changed due to the COVID-19 situation (61.3 and 60.3% respectively). For those who reported a change, 25.1% of members and 24.3% of the general population reported a decrease, whilst 13.6% members and 15.4% general population reported an increase in fruit intake.

***Vegetable intake*** There was an overall significant time effect on vegetable intake for SW members (F (2.78,558.91) = 34.467, *p* < 0.05). Post-hoc tests showed that vegetable intake increased significantly after joining (2.5 ± 1.31 vs 3.5 ± 1.37 portions; T0 vs T1, *p* < 0.001) and remaining higher at 3 (3.4 ± 1.38 portions, *p* < 0.001) and 6 months (3.2 ± 1.40 portions, *p* < 0.001).

There was no difference in vegetable intake between samples of the general population at each time point (F (2,3545) = 0.727, *p* > 0.05). Vegetable intake was significantly greater for SW members compared to the general population samples at each time point (*p* < 0.05).

Most SW members (71.1%) and the general population (64.8%) reported no change in vegetable consumption due to the COVID-19 situation. Where there was a change, 18.9% of members reported eating fewer vegetables and 10% reported eating more. For the general population 16.6% reported a decrease and 18.5% reported an increase in vegetable intake.

***Sugary drinks intake*** Frequency of sugary drink consumption by the SW members changed significantly over time points (F (1.95,386.48) = 15.79, *p* < 0.001). Post-hoc tests showed that members significantly decreased their consumption of sugary drinks after joining (1.5 ± 1.01 vs 1.2 ± 0.52; T0 vs T1, *p* < 0.01). This intake remained stable, with no significant changes at further time points.

For the general population samples, intake varied over time (F (2:3545) = 3.338, *p* < 0.05) and was lower at T2 than T1 (1.7 ± 1.16 vs 1.8 ± 1.21; *p* < 0.01) and increased between T2 and T3 (1.8 ± 1.21, *p* < 0.05). SW members were having sugary drinks significantly less often than the general population at each time point (*p* < 0.05).

The majority of both SW members (89.9%) and the general population (74.1%) reported no change in their intake of sugary drinks due to the COVID-19 situation. The general population were more likely to have reported a change, with a greater proportion reporting both an increase in intake (12.7% vs 5.5%, general population vs members, *p* < 0.01) and a decrease (13.2% vs 4.5%, general population vs members, *p* < 0.001).

***Wholegrains intake*** The frequency of SW members’ wholegrain consumption changed significantly over time (F (2.74,550.71) = 6.357, *p* < 0.001). Average frequency of intake increased after joining (2.4 ± 1.26 vs 2.6 ± 1.14; T0 vs T1, *p* < 0.01) and remained higher at the 3 (2.7 ± 1.18; T2) and 6 month points (2.6 ± 1.14; T3).

There was no overall difference in wholegrain intake for the general population across time points (F (2,3545) = 1.112, *p* > 0.05). The general population sample were eating wholegrains less often than SW members at T1 (*p* < 0.001) and 3 months (*p* < 0.001) but there was no difference at the 6-month follow up point.

The majority of both SW members (83.1%) and the general population (82.4%) reported no change in the frequency of wholegrain consumption due to the COVID-19 situation. 10.0% of members reported a decrease and 7% reported an increase in how often they consumed them, which was similar to the general population, with 7.4% reporting a decrease and 10.2% reporting an increase.

***Fatty food intake*** The frequency of fatty food intake by SW members differed over time (F (2.78,581) = 238.05, *p* < 0.001) with post-hoc tests showing that frequency of fatty food intake dropped significantly after joining (3.5 ± 1.18 vs 1.5 ± 0.83; T0 vs T1, *p* < 0.001). Intake then increased slightly at T2 (1.5 ± 0.83 vs 1.7 ± 0.07;T1 vs T2, *p* < 0.05) and again by T3 (2.0 ± 1.13; *p* < 0.001). Intake remained lower at the 6-month point (T3) compared to levels prior to joining (T0, *p* < 0.001).

Frequency of fatty food intake for the general population samples varied over time (F (2,3545) = 7.188, *p* < 0.001) and was significantly lower at T2 compared to T1 (3.0 ± 1.11 vs 2.9 ± 1.09; T1 vs T2, *p* < 0.001) which then increased at T3 (3.0 ± 1.12; *p* < 0.05) meaning it was no different from that of the sample at T1 or the SW members at T0 (before joining SW). The frequency of fatty food intake for the general population samples remained higher than the SW members at T1, T2 and T3 (*p* < 0.001).

Around two thirds of SW members (61.0%) and the general population (65%) reported no change in how often they were eating fatty foods due to the COVID-19 situation. For members, 12% reported a decrease and 27% reported an increase in how often they were eating fatty foods. For the general population, 17.3% reported a decrease and 17.7% reported an increase. Whilst a greater percentage of members reported an increase in how often they were eating fatty foods at this time, the quantitative data (Fig. 2) shows that members were still eating fatty foods less often than the general population at each time point (*p* < 0.001).

***Sugary food intake*** There was an overall significant difference in how often SW members were consuming sugary food intake across time points (F (3,627) = 186.775, *p* < 0.001). Post-hoc tests showed, after joining, members started eating sugary foods less often (4.0 ± 1.16 vs 1.8 ± 1.13; T0 vs T1, *p* < 0.001). The frequency of sugary food intake increased at 3 months (2.6 ± 1.29; T2, *p* < 0.001) and again at 6 months (2.9 ± 1.36; T3, *p* < 0.001) although they were still being eaten less often than they were prior to joining.

Frequency of intake of sugary foods differed over time for the general population samples (F (2,3545) = 6.146, *p* < 0.001). Data from the T1 (3.4 ± 1.22) and T2 (3.4 ± 1.23) showed no significant difference in how often the general population were consuming sugary foods. The intake increased significantly at T3 (3.6 ± 1.20) compared to T1 (*p* < 0.01) and T2 (*p* < 0.001) and remained significantly greater than the intake of SW members at all time points (*p* < 0.05).

Around half (49.5%) of SW members and the general population (52%) said the COVID-19 situation had not affected how often they were eating sugary foods. A proportion of both groups, 41.5% for members and 34.2% of the general population, reported an increase in frequency of eating sugary foods, while only 9% of members and 13.8% of the general population reported a decrease. While 41.5% of members reported they were eating sugary foods more often due to the COVID-19 situation, they were still eating sugary foods significantly less often than the general population at all time points (*p* < 0.05).

***Cooking from scratch*** There was a difference in how often members cooked from scratch over the time points (F (2.32,485.08) = 60.166, *p* < 0.001) with significant increases occurring between T0 and T1 (4.0 ± 1.08 vs 4.7 ± 0.63;T0 vs T1, *p* < 0.001). Data remained stable and did not differ significantly at subsequent time points and members remained cooking from scratch significantly more often than at T0.

There was some variation in how often the general population were cooking from scratch over time (F (2,3545) = 3.236, *p* < 0.05). The sample at the 6-month survey were cooking from scratch significantly more often than those sampled at T1 (3.9 ± 1.13 vs 4 ± 1.14, T1 vs T3, *p* > 0.05) but there were no differences at other time points.

Around half of SW members (58%) and the general population (49.6%) reported the COVID-19 situation had not affected how often they cooked from scratch. However, in those who reported a change, the majority said they were cooking from scratch more often (32.5% of members and 46.5% of the general population). A much smaller proportion of members (9.5%) and the general population (3.9%) reported cooking from scratch less often. Even though a greater proportion of the general population reported an increase in cooking from scratch due to the COVID-19 situation, the longitudinal data shows that SW members were cooking from scratch significantly more often at each time point (*p* < 0.05).

***Alcohol intak***e SW members’ alcohol consumption varied over time (X^2^ (3)=39.2, *p* < 0.001) with post-hoc tests showing that members reduced their average weekly unit consumption soon after joining (15.7 ± 10.20 vs 12.0 ± 10.50 units/wk.; T0 vs T1, *p* < 0.001) and this decreased further at the 3-month point (8.9 ± 8.56 vs 12.0 ± 10.50 units/wk.; T2 vs T1, *p* < 0.001). There was an increase in alcohol intake from 3 to 6 months, although average weekly units were still significantly lower than before joining (12.7 ± 9.86 vs 15.7 ± 10.20 units/wk.; T3 vs T0, *p* < 0.001).

For the general population samples, alcohol intake differed over time (x^2^ (2)=11.7, *p* < 0.001). There was no significant difference in mean intake of alcohol units per week between the samples at the first and second surveys (16.2 ± 26.2 vs 14.3 ± 19.6 units/wk.; T1 vs T2, _adj_*p* = 0.104). However, the general population sample at 6-months had lower alcohol consumption on average compared to the sample at T1 (14.2 ± 19.90 vs 16.2 ± 26.2; T3 vs T1, *p* < 0.01). The general population consumed more units of alcohol per week than SW members at T1 and T2 (*p* < 0.05).

The majority of SW members (66.7%) and the general population (72.4%) reported that the COVID-19 situation had not affected their alcohol intake. However, 27.7% of members and 16.3% of the general population reported an increase, whilst 5.6% of members and 11.3% of the general population reported a decrease in alcohol intake. Despite a greater proportion of members reporting that their intake of alcohol had increased due to the COVID-19 situation, the results above show members had already significantly reduced their alcohol intake after joining, and at this time were still drinking less than they were before they joined, and were below the recommended guidelines of a maximum of 14 units per week.

#### Physical activity levels (Fig. 3)

Hours of moderate intensity physical activity per week changed over time (F (1.63,213.17) = 12.925, *p* < 0.001), with SW members increasing their activity levels within the first 3 months (3.9 ± 4.74 vs 6.5 ± 11.7 h/wk.; T1 vs T2, *p* < 0.001) and again from 3 months to 6 months (6.5 ± 11.7vs 9.0 ± 8.56 h/wk.; T2 vs T3, *p* < 0.001).

**Fig. 3:**
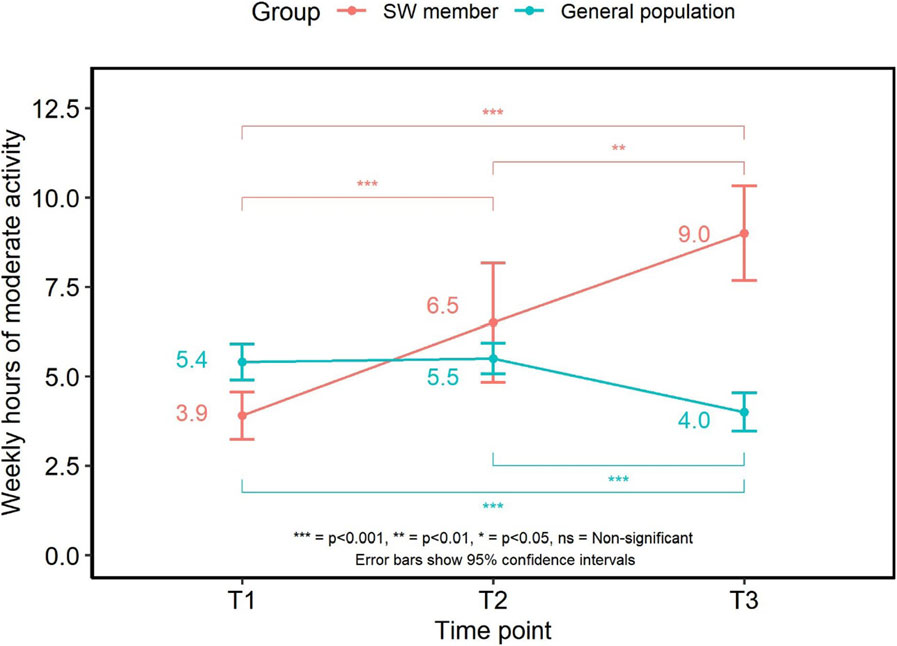
Weekly hours of moderate intensity activity for SW members and general population sample

There was variation in hours of weekly moderate intensity activity over time for the general population (F (2,3089) = 6.951, *p* < 0.001). Average weekly activity levels did not differ significantly between the first two survey samples (5.4 ± 10.1 vs 5.5 ± 6.95 h/wk.; T1 vs T2), but levels were significantly lower at 6 months (4.0 ± 6.35; T3) compared to the samples at T1 and T2 (*p* < 0.001).

When asked whether the COVID-19 situation had affected activity levels, there was a spread across the responses. The majority of the general population (42.5%) and 38.7% of SW members reported no change in their activity levels whilst 40.5% of the general population and 36.2% of members reported a decrease in activity. A greater proportion of SW members reported their activity levels had increased compared with the general population (25.2% vs 17.0%; members vs general population, *p* < 0.05).

### Well-being

Well-being changed significantly over time for members (F (1.9417.21) = 130.708, *p* < 0.001) increasing between the baseline and 3-month survey points (22.2 ± 2.93 vs 29.4 ± 6.20; T1 vs T2). At the 6-month survey, well-being scores had decreased compared to that at the 3 month point (27.8 ± 6.73; T3, *p* < 0.001) but remained higher than that at T1 (27.8 ± 6.73 vs 22.2 ± 2.93; T3 vs T1, *p* < 0.001).

**Fig. 4:**
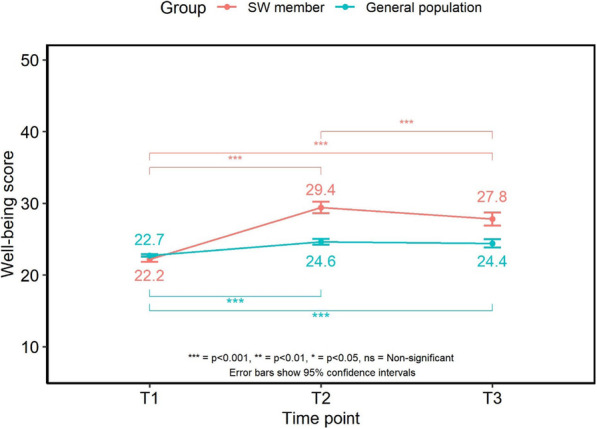
Mean scores and 95% confidence intervals for well-being over time

Well-being changed over time for the general population samples (F (2,3545) = 47.374, *p* < 0.001), which was greater within the 3-month sample compared to the sample at the first survey (24.6 ± 7.52 vs 22.7 ± 3.93; T2 vs T1, *p* < 0.001). Well-being scores at the 6-month survey did not differ compared to the 3-month survey (*p* > 0.04) and were still higher than data from the first survey (24.4 ± 7.23 vs 22.7 ± 3.93; T3 vs T1, *p* < 0.001). Well-being scores were significantly higher for SW members at T2 (*p* < 0.001) and T3 (*p* < 0.001) (Fig. 4).

Around half (52%) of SW members and the general population (50.9%) reported their mood had not been affected by the COVID-19 situation. Just over a third of members (36.3%) reported their mood had decreased compared to 42.4% of the general population but this was not significantly different (*p* > 0.1).

### Qualitative responses about the impact of COVID-19 on weight management

In terms of how easy or difficult people found managing their weight during the COVID-19 situation, responses from SW members and general population were similar. In total, 59.3% of members and 64.5% of the general population reported finding managing their weight ‘difficult’ during lockdown.

When asked to explain why they had found managing their weight easy or difficult during this time, there was a wide range of responses from both members and the general population with some saying they had found it easy to others reporting various difficulties. After generating the initial codes, five broad themes were identified following the search, review and defining procedures (Table 2) with changes to routine an underlying theme to all five themes. Some respondents found it was easy to maintain a healthy lifestyle whilst others reported difficulties in maintaining a healthy diet due to access to certain foods, temptation to snack more because of boredom, comfort eating and drinking more alcohol. Similarly, there was a mix of responses around physical activity with some reporting they were now exercising and moving around more while others reported being less active. Stress and anxiety were reported to negatively affect eating behaviours too.

**Table 2:** underlying and related sub-themes emerging when respondents were asked to explain why managing weight was easy or difficult during the COVID-19 affected period

Underlying theme: Changes to routine	
Key theme	Related sub-themes
Harder to access healthier food options (Barrier)	Shopping less frequently; limited availability; fresh produce not lasting in-between shops; limited delivery services available; reduced income
More time at home (Barrier)	Eating through boredom; lack of routine; greater accessibility to food; increased snacking; drinking more alcohol
Emotional impact of lockdown (Barrier)	Anxiety; worry and stress; feeling down/low; comfort eating; reduced income; health related issues; drinking more alcohol
Changes to physical activity levels (Barrier & Enabler)	Less exercise; no gyms; no work-related exercise; sedentary behaviours; no facilities; need to shield; more time to exercise
Maintenance of healthy habits (Enabler)	Coping strategies; more time to plan; cooking more from scratch; not socialising as often; motivation

Overall, there were more **barriers** than **enablers** to maintaining healthier lifestyle changes because of the COVID related situation.

#### Harder to access healthier food options

Many reported difficulties in shopping and accessing certain foods, with usual healthier alternatives not being available. There were reports of difficulties obtaining fresh fruit and vegetables and healthier options such as skimmed milk. Difficulties in accessing shops or being able to get online delivery slots (including difficulties financing the minimum spend) were reported. Shopping less frequently and making a larger shop or being dependent on others were mentioned as making it more difficult to have a regular supply of fresh foods particularly vegetables and salad ingredients due to their shelf life. Some also mentioned the larger shops meant that there were more tempting snack foods available in the house. Furlough and a reduction in household income was mentioned as impacting on shopping habits, making it more difficult to shop as often and have healthy foods in the house.

‘*Fresh fruit and veg sometimes dont turn up with order, but cookies always do*’ (General population).

‘*Unable to get deliveries of some foods that are helpful for a healthy diet. Supermarkets substituting unhelpful alternatives’*. (SW member).

‘*Not doing my own shopping relying on others to bring food in from shops I don’t use normally’*. (SW member).

‘*It’s harder to keep a supply of fresh fruit veg and salad without going to the supermarket every 3-4 days’* (General population).

‘*Because my husband has been furloughed from his job for only 80% of his salary, we can’t afford to get as much shopping as we would get before. We aren’t able to get out as I am having to self isolate due to underlying health conditions’.* (General population).

Some mentioned the need to shield due to health conditions making it difficult to access healthy foods. Keyworkers, particularly those working in the NHS, reported finding it difficult to find time to shop for food, a lack of healthy foods available during long shifts, feeling exhausted and grabbing snacks more often rather than having a time for a meal.

‘*Working shifts in ICU in hospital, little time to shop, unhealthy food more available and exhausted’* (SW member).

#### More time at home

Many respondents, both the general population and SW members, reported snacking more frequently on less healthy foods due to being at home, boredom and being out of a normal routine or lack of structure to the day. Being at home during the day with children who are snacking was mentioned as increasing temptation to join in with snacks. Spending more time at home was also reported to increase temptation to spend more time in sedentary activities such as watching TV and increasing temptation to more alcohol. Some mentioned baking more often to occupy children and grandchildren which meant they felt they now had more higher fat and sugar foods to eat.

‘*Because of too much snacking out of boredom. Also, the children always munching on something like crisps and sweets and it makes me want to join them’*. (General population).

‘*Having a lot of extra time, boredom and being at home with food in the cupboards/fridge I have found it difficult to stay on plan but I am managing it’*. (SW member).

‘*I am stuffing my mouth with sweet stuff - I need to lose weight I have put on a stone if not more’* (General population).

‘*More time at home and am not working. Drinking more alcohol which gives you the alcohol munchies. Bit of a feeling of the school holidays’* (SW member).

#### Emotional impact of lockdown

The emotional impact of lockdown was clear amongst SW members and the general population, with many reporting feeling stressed, worried, anxious and/or low. Some reported that there were too many things to worry about to think about managing their weight. Key workers mentioned not being able to prioritise healthy eating, activity or weight management at this time. There was mention of poor mental health and ‘having no interest in anything else including eating healthily’. Many reported they were comfort eating because of their mood and some said they were using food and alcohol as coping mechanisms.

‘*A depressing time and can’t contemplate watching what I eat or drink’*. (General population).

‘*My mental health is suffering and I have no interest in anything including eating healthy’*. (SW member).

‘*Working in the NHS has been stressful and sometimes it’s easy to grab a snack rather than a meal, plus sitting around more had led to comfort eating’*. (SW member).

‘*Anxiety makes me eat as I eat for comfort. Always the wrong foods - sugary and sweet’*. (SW member).

‘*I am lacking in the motivation to do anything; I had just recovered from depression and now I have no real reason to leave the house. I used to walk all the time and do Yoga and I just cannot be bothered’*. (General population).

#### Changes to physical activity levels

Responses were mixed in terms of physical activity; while some felt that lockdown had encouraged them to be more active, the majority felt that lockdown had prevented them from being active. The main barrier to physical activity was the closure of gyms, leisure centres and exercise classes. Those who had previously exercised at the gym or engaged in swimming, team sports or exercise classes noted a decrease in activity levels. Underlying health conditions, the need to shield and having symptoms of the virus such as breathing difficulties and fatigue were mentioned by a few respondents as a cause of being less active. Some reported that they had more time to get outside to walk or cycle and some talked about engaging in online exercise classes. Being furloughed for some meant that they had more time to exercise, while for others meant that they no longer had the daily walk or cycle to and from work. Some respondents mentioned that working from home meant they were sat down a lot more and not moving around as much as they would normally do in their job. For others, not being in the office meant they now spent less time sitting.

‘*I cannot do all the activities like swimming, the gym, cinema, theatre, Meetup walks, socialise with friends, look after my grandkids that I normally do on a weekly basis… Not as physically active…*’ (SW member).

‘*Can’t exercise as much as I would like. No longer walking as part of my journey home from work’*. (General population).

‘*Can’t go to gym, don’t have space or equipment to exercise at home properly and lack of motivation’* (General population).

‘*Time to be more active and taking bike rides with my son. Not working so sitting down a lot less’*. (SW member).

‘*Furloughed from work so have more time to exercise’*. (General population).

#### Maintenance of healthy habits

This theme, an enabling theme, was the least common for both groups of respondents, but some did express a commitment to maintaining healthy habits and finding alternative forms of exercise. Some found that they had more time and were able to go for a daily walk which they would not have done before lockdown.

‘*I was advised to lose weight for health reasons which along with my consultant at SW gave me the inspiration to do so. I am motivated and determined about this and have no intentions of regressing’*. (SW member).

‘*Plenty of time to go walking and plan meals, shorter working hours means i can be home earlier and take time over making dinners’*. (General population).

‘*I have had more time to cook, mostly from scratch & more time to exercise (plus gardening, cleaning, sorting)*’. (SW member).

*‘Easier, cant get certain foods so not much to snack on’*. (General population).

There was mention of not eating out or eating takeaways as often and cooking from scratch more which were highlighted as making it easier to now eat more healthily and manage weight. Some also reported socialising less meant they were now drinking less alcohol.

‘*No more coffee and cake in cafes!*’ (General population).

Some of the SW members who had joined six months ago reported having coping strategies in place.

‘*Daily diary and bought the best scales to match SW*’ (SW member).

‘*It’s always difficult! But just belonging is enough to keep me in check, even if I am actually losing very little’*. (SW member).

#### Change to normal routine – other factors

All five key themes were related to the changes in routine experienced during lockdown. There were a few other areas cited which do not sit within the key themes but are also related to the changes in routine and which may affect dietary habits and weight status. These areas include the impact on sleeping patterns, living arrangements and the consequence of the SW groups being delivered virtually rather than in-person.

‘*I am sleeping badly. I am awake for longer than usual so I am eating more, causing weight gain’*. (General population).

‘*Because I’m not in my normal routine and I find it easier to be healthy in a routine’*. (SW member).

## Discussion

This mixed-methods study surveyed adults who had joined a weight management programme, (Slimming World, SW), six months previously and a representative sample from the general population. Given that the six-month time point corresponded with the first UK lockdown due to COVID-19, the study team were curious to investigate how the related changes may have impacted on health-related behaviours, well-being and the ability to manage weight. It was predicted that these behaviours would have been negatively affected. Over half of both groups of respondents said that they had found it more challenging to manage their weight during the COVID-19 lockdown period.

The SW respondents were predominantly female with a mean BMI placing them in the obese category. Around 12% of the original new SW members completed the six month survey. They had been able to maintain a mean weight loss of almost 8% at six months with no mean absolute weight gain observed and with this group of 222 adults achieving a mean BMI reduction of 3 kg/m^2^. The data collected suggests that a number of changes to dietary behaviours were made within the first few weeks after joining the programme, many of which were maintained over the following three months, which were likely to have contributed to the weight loss observed. These dietary changes included eating more fruits, vegetables, eating fewer fatty and sugary drinks/foods, eating more wholegrains, cooking more from scratch and cutting down on alcohol intake. Increases were seen in physical activity levels which may have also contributed to the maintained weight loss. Levels continued to increase 3–6 months after joining.

The representative general population respondents had a mean BMI which would have placed them in the overweight towards obese category and which did not differ between samples. They were recruited as not currently accessing weight management support from a commercial provider. The baseline dietary behaviours of the general population were very similar to the dietary habits of the SW members before they joined the weight management programme suggesting that the member behaviour was very similar to the general population sample and that the weight loss achieved and maintained by SW members was as a consequence of sustained behaviour change. Furthermore, the dietary habits of the general population samples did not differ between the first two time points apart from alcohol intake. There were no reported differences in income levels between the two groups. Given these similarities between the groups, the data provides an insight into how people accessing a weight management support programme before lockdown were able to respond to the dietary challenges faced during lockdown in contrast to the cross-section of a representative sample from the wider population who had not received this support. SW members were able to maintain most of the dietary changes they had already made, despite the challenges faced. A notable proportion of both members and the general population reported increasing the frequency of intake of both sugary and fatty foods at this time and this is mirrored by the findings of similar research looking at the impact of the UK lockdown on eating behaviours [8]. This was also reflected by the quantitative data. However, due to already having reduced the frequency of fatty and sugary food intake since joining Slimming World, members were still consuming less than they had been previously, and were consuming sugary and fatty foods less often than the general population during lockdown. Whilst there was some reporting of alcohol intake increasing due to the COVID-19 situation, average intakes of the general population did not differ at this time, whereas the quantitative data for members indicated a significant increase. However, again due to the significant reductions in alcohol intake made by members within the first three months of their membership, even though their weekly units increased during the COVID-19 situation, they were still drinking below the government recommendations, and less than before they joined Slimming World.

When asked whether activity levels had changed due to the COVID-19 situation, there was a fairly even spread across responses, with both members and the general population reporting increases, decreases and no change in activity. Interestingly, clearer patterns emerged from the longitudinal data. There were differences in physical activity behaviour between the two groups with the SW members being less active before they joined the programme compared to the general population samples. Whilst the general population sample substantially decreased the time they spent engaged in physical activity during lockdown, the SW members, on average, continued to increase the amount of time they spent being physically active, suggesting that the behaviour change they had already initiated helped them maintain this healthy routine and prevent the decrease in activity seen in the general population.

The qualitative data suggested that changes to routine during the lockdown period had a major impact and contributed to the key themes observed when respondents were asked if and why managing their weight was easy or difficult during the lockdown period. Clearly some respondents, particularly the SW members, had developed different coping strategies, some respondents found the extra time available made it easier to find time to exercise or to cook meals from scratch whilst others expressed the fact that because they were able to socialise less they were not meeting up with friends for coffee and cake.

However far more respondents from both groups reported the difficulties they had encountered with a number of barriers preventing them maintaining healthier habits. Overwhelmingly many people said that they found it harder to access healthier food options and this was for a number of reasons. The changes to the food environment resulting from the pandemic made access to routinely consumed foods more challenging with guidance early on for people to limit visits to supermarkets to once per week. This coupled with many stores having reduced availability and people having reduced geographic mobility due to isolation, meant accessing fresh produce became difficult. Despite this, SW members managed to maintain their increased consumption of fruit and vegetables during lockdown, which was significantly greater than that of the general population. Some people expressed the fact that their incomes had been reduced as a consequence of the pandemic and this limited their budget for food expenditure. Concerns about financial stability may have added to the food insecurity experienced by many during lockdown [9, 10].

Another contributing factor to the dietary habits resulting from lockdown which made weight management more challenging was the strong ‘eating through boredom’ theme. Again, changes to routine, being at home more with greater accessibility to certain foods led to a greater perceived level of snacking and snacking on foods that they would not normally choose. Marrying up the quantitative and qualitative data, both groups did increase their intake of sugary foods during the lockdown period and this may have been due to eating more through boredom and snacking on foods like chocolate, cakes and confectionary. Indeed some people said that because they had more time on their hands, they were baking more.

Across the UK, people’s level of anxiety increased as a consequence of COVID-19. There was and there still is a lot of uncertainty [11]. For many this means emotional eating and eating foods and drink they would not normally choose [12]. The acknowledgement that obesity increases the severity of symptoms and worsens treatment outcomes may have led to increasing anxiety levels for those people with high BMIs [8]. In the present study, over a third of both members and the general population reported that their overall mood had decreased due to the COVID-19 situation. This is reflected in the longitudinal data, as the overall well-being score of members had significantly reduced, however it remained higher than that of the general population. Many people from both study groups expressed that stress, worry and anxiety were barriers that had made weight management more challenging. Financial challenges added to their stress as did health-related issues. They recognised they were emotional eaters and comfort eating on sugary foods or drinking more alcohol as a consequence of their emotions. Some recognised that whilst they had anxiety and other problems to worry about initially, they then needed to have strategies in place to return to their healthier dietary practices. The finding that for some people, the COVID-19 lockdowns are a time of high risk for over-eating has also been found in a recent online survey of a mainly UK based adult population where almost half reported an increased food intake although with a large individual variability in the craving of high-energy dense sweet and savoury foods [13]. Similarly, the anxiety we report has also been explored in an online survey (*n* = 264) of perceived changes in eating, exercise, and body image related to mental health during lockdown within the UK. Again, there were large individual differences in the perceived changes with women more likely than men to report increasing struggles with regulating eating, their preoccupation with food and worsening body image [14].

Early government guidance allowed people to just go out once a day for physical activity with walking positively encouraged. However, many people do not have pleasant and safe parks or green spaces in order to achieve this and because of the need to shield, felt too vulnerable to go outdoors. Gyms, swimming pools and leisure centres were closed at the time of the survey. Thus, whilst some may have been able to have increased their physical activity levels, others will have become more sedentary. This was certainly the findings of the qualitative work where some respondents said that lack of facilities and motivation were barriers that contributed to reduced physical activity levels whilst others said that having more time available because of being furloughed, enabled them more time to be active. Whilst the SW members engaged in less physical activity before joining the programme compared to the general population representative sample, they were still able to further increase their activity levels during lockdown. In contrast the general population did decrease their levels. This contrast may have been due to the focus on increasing activity levels as part of the SW programme during the lockdown where the delivery changed to virtual groups with the use of social media platforms to extend the support offered. A specific campaign to help members be active at home was launched.

The changes to routine during lockdown may have affected sleeping patterns. Quantitative data was not collected about sleeping habits which may have been of interest and a limitation of the current study. Certainly for some people reduced sleep can have an impact on dietary behaviours with some people eating more as a consequence [15].

Longitudinal data is only available for the SW members with representative samples of the general population recruited at each time point. Whilst a richer understanding of the general population data would be obtained using a longitudinal design the primary aim of this study was to understand the changes SW members made over time and how their health and behaviours compared to samples of the general population. The consistency in measures between the general population samples suggest that these data do offer a practical insight into behaviours that are typical within the wider general population and serve as a representative comparison with SW members. The study is limited by the low response rate at the 6 month point with only 222 of the original 1884 SW members completing the surveys at all three time points, of which this subsample were older and may not be totally representative of the overall SW membership. In addition the use of self-reported data may have led to bias in the reporting of weight, lifestyle and well-being changes although given the remote nature of this research self-reported measures were the only viable means of collecting such data. Being a member of SW may have led to some reporting bias through the sub conscious effect of being part of an organisation and perceived demand characteristics alongside impression management. We did not assess levels of food insecurity at baseline or changes in levels of food insecurity at subsequent time-points although the median annual household income levels were the same for the study groups and the general population at each time-point. However, we appreciate that levels of food insecurity may be an important variable to consider in future research and may limit the wider application of our findings. Similarly, underlying health conditions may have also influenced the results but were not considered in this study. A further limitation is that some error may have been introduced through the use of parametric analysis for the Likert data.

However, using the mixed-methods approach allows the ability to verify the findings through the use of the quantitative and qualitative data and indeed good verification was reported for both groups. The reported findings from this study are also in-line with other observations of the impact of the COVID-19 situation on health-related behaviours [6, 8, 13, 14, 16]. A further limitation could be that one would expect to see some return to original health-related behaviours in people accessing weight management support. However the findings reported at three months after joining SW are very similar to the behavioural changes reported and sustained in a previous study of the health-related changes achieved and their contribution to weight management at 12 months [17, 18].

In conclusion, this study found that the COVID-19 lockdown period affected adults who had been accessing weight management support in a similar way to the general population with both groups finding weight management more challenging due to the changes in routine and it being harder to access healthier foods. It found people’s eating and drinking habits were affected because of spending more time at home, boredom and emotional eating because of the added stress and anxiety. However, there were some who reported that the extra time afforded to them by the lockdown enabled them to plan better, cook from scratch more and allowed them to fit physical activity into their routines, suggesting they had developed strategies to help protect their weight.

Helping people cope with set-backs throughout their weight loss journey may help them establish the skills needed to cope with situations such as the COVID-19 lockdown. Overall, SW members were actually able to increase their activity levels, whilst lockdown did seem to negatively impact on the physical activity levels of the general population. SW members showed a level of resilience and were able to maintain most of the positive dietary changes that they had made since joining, and during the COVID-19 situation were still reporting improvements in all dietary behaviours measured, with healthier scores than the general population, and maintained their earlier weight loss.

## Data Availability

Data is available on request from the corresponding author. The survey is available via Supplementary material.
